# Proof-of-concept study demonstrating the pathogenicity of affinity-purified IgG antibodies directed to domain I of β_2_-glycoprotein I in a mouse model of anti-phospholipid antibody-induced thrombosis

**DOI:** 10.1093/rheumatology/keu360

**Published:** 2014-09-30

**Authors:** Charis Pericleous, Patricia Ruiz-Limón, Zurina Romay-Penabad, Ana Carrera Marín, Acely Garza-Garcia, Lucy Murfitt, Paul C. Driscoll, David S. Latchman, David A. Isenberg, Ian Giles, Yiannis Ioannou, Anisur Rahman, Silvia S. Pierangeli

**Affiliations:** ^1^Centre for Rheumatology Research, Division of Medicine, University College London, London, UK, ^2^Division of Rheumatology, Department of Internal Medicine, University of Texas Medical Branch, Galveston, TX, USA, ^3^Structural Biology, Medical Research Council National Institute for Medical Research and ^4^Arthritis Research UK Centre for Adolescent Rheumatology, University College London Hospital and Great Ormond Street Hospital, London, UK.

**Keywords:** anti-phospholipid syndrome, anti-phospholipid antibodies, β2-glycoprotein I, domain I, venous thrombosis, mouse model

## Abstract

**Objective.** IgG aPL against domain I of β_2_-glycoprotein I (β_2_GPI) [anti-DI (aDI)] is associated with the pathogenesis of APS, an autoimmune disease defined by thrombosis and pregnancy morbidity. To date, however, no study has demonstrated direct pathogenicity of IgG aDI *in vivo*. In this proof-of-concept study, we designed a novel system to affinity purify polyclonal aDI aPL in order to assess its prothrombotic ability in a well-characterized mouse microcirculation model for APS.

**Methods.** Two polyclonal IgG fractions were isolated from serum of a patient with APS, both with high aPL activity but differing in aDI activity (aDI-rich and aDI-poor). These IgG fractions were tested for their pathogenic ability in an *in vivo* mouse model of thrombosis. Male CD1 mice were injected intraperitoneally with either aDI-rich or aDI-poor IgG; as a control, IgG isolated from healthy serum was used. A pinch injury was applied to the right femoral vein and thrombus dynamics and tissue factor activity in isolated tissue were evaluated.

**Results.** Both aDI-rich and aDI-poor IgG retained aCL and anti-β_2_GPI activity, while only aDI-rich IgG displayed high aDI activity, as defined by our in-house cut-offs for positivity in each assay. aDI-rich IgG induced significantly larger thrombi *in vivo* compared with aDI-poor IgG (*P* < 0.0001). Similarly, aDI-rich IgG significantly enhanced the procoagulant activity of carotid artery endothelium and peritoneal macrophages isolated from experimental animals (*P* < 0.01).

**Conclusion.** These data directly demonstrate that the ability to cause thrombosis *in vivo* is concentrated in the aDI fraction of aPL.

## Introduction

APS is a systemic autoimmune disease caused by circulating aPL [1]. APS is a major cause of acquired hypercoagulability and recurrent pregnancy loss in the general population [2].

Antibodies to phospholipid (PL), and to serine protease clotting factors and PL-binding proteins circulate in the blood of patients with APS. The strongest evidence for pathogenicity relates to antibodies against the PL-binding protein β_2_-glycoprotein I [anti-β_2_GPI (aβ_2_GPI)] [3].

β_2_GPI has five domains (DI–DV). Although antibodies directed against all of these domains have been reported in patients with APS, anti-DI antibodies (aDI) are most closely linked to pathogenicity. We [4] and other groups [5–8] have shown that circulating levels of IgG aDI are elevated in patients with APS compared with healthy and disease controls.

We previously demonstrated that human recombinant DI abrogated the thrombogenic ability of polyclonal human IgG purified from a patient with APS in our well-characterized *in vivo* mouse model of thrombotic APS [9], while a human monoclonal IgG aPL, IS4, both bound DI [10] and enhanced clot formation in the same model [11, 12]. Critically, however, no study has shown that affinity-purified IgG aDI from patients with APS has a direct prothrombotic effect *in vivo*. Therefore this study was designed to test the hypothesis that the ability to cause thrombosis in mice is concentrated in the aDI fraction of polyclonal IgG derived from serum of a patient with APS.

## Patients and methods

### Affinity purification of anti-DI antibodies

Serum was obtained from a female patient with APS. Polyclonal IgG from this patient has previously been shown to cause thrombosis in our mouse model [13]. Patient serum was fractionated into aDI-rich and aDI-poor fractions as follows: human recombinant hexahistidine-tagged DI was produced and purified as described previously [10, 14]. Immobilized-nickel resin beads (Promega, Madison, WI, USA) were equilibrated with PBS (pH 7.4), followed by incubation with purified DI (50 μg DI/100 μl resin) for 30 min. DI-coupled beads were incubated with patient serum (1 ml serum/100 μl resin) for 1 h. The beads were then spun, the flow-through was collected (aDI-poor fraction) and beads were washed with PBS. Captured aDI antibodies (aDI-rich fraction) were eluted from the beads with 0.1 M glycine (pH 2.7), concentrated and dialysed in PBS (Amicon Pro centrifugal devices, Millipore, Billerica, MA, USA).

### Human polyclonal IgG purification

IgG was purified from both aDI-rich and aDI-poor fractions using protein G chromatography (ThermoScientific Pierce, Rockford, IL, USA). Pooled sera from 10 healthy donors who tested negative for aPL [normal human serum (NHS)] were used as a source of control IgG. aDI-rich IgG, aDI-poor IgG and NHS-IgG were concentrated and dialysed in PBS and were confirmed to be free of endotoxin by the limulus amoebocyte lysate assay (Sigma, St Louis, MO USA). All research subjects who donated serum signed informed consent according to the Declaration of Helsinki and the study was approved by the Institutional Review Board of the University of Texas Medical Branch.

### Immunological characterization of serum and purified polyclonal IgG

IgG aCL, aβ_2_GPI and aDI activity were measured in human serum (diluted 1:50), purified human IgG (aDI-rich, aDI-poor and IgG purified from unfractionated serum, all tested at 100 μg/ml) and mouse serum (diluted 1:10) using direct ELISA as described previously [4]. Total human IgG circulating in mouse serum was measured by ELISA [12].

To determine the avidity of purified IgG to CL, β_2_GPI or DI, we tested IgG (at 100 µg/ml) in the presence of increasing salt concentrations (range 0.15–4 M NaCl, where PBS has 0.15 M NaCl).

aCL activity was defined as IgG phospholipid units (GPLU), using commercially sourced calibrators (Louisville aPL Diagnostics, Seabrook, TX, USA; activity range <16 to >96 GPLU), while aβ_2_GPI and aDI activity were defined as IgG β_2_GPI units (GBU) and DI units (GDIU), respectively, using in-house calibrators (activity range <3.1 to >100 for both tests). Based on sera from a population of 200 healthy subjects, our cut-offs for aPL positivity in these assays are (defined as the mean + 3 s.d. of the activity of healthy subjects) 17 GPLU, 8 GBU, and 10 GDIU.

### In vivo induction of thrombosis and analysis of thrombus dynamics

Five to 10 male CD1 mice (Charles River Laboratories, Cambridge, MA, USA) per group were injected intraperitoneally twice (at time 0 and 48 h later) with either 100 µg of aDI-rich or aDI-poor IgG (*n* = 10/group) or NHS-IgG (*n* = 5). Mice were anaesthetized 72 h after the first injection. The right femoral vein was exposed and pinched using a standard pressure to induce injury. Thrombus dynamics were assessed as described previously [9, 15, 16]. Serum was obtained for ELISA after the surgical procedure.

### Tissue factor activity in carotid homogenates and peritoneal macrophages

At the end of the surgical procedures, uninjured carotids were placed into Tris-buffered saline–0.01% Triton X-100 buffer containing heparin. Peritoneal macrophages were collected by flushing the peritoneal cavity with PBS for 5 min. After sonication, tissue factor (TF) activity was determined using a commercial chromogenic assay (Human Tissue Factor Activity Kit, AssayPro, St Charles, MO, USA). TF activity data were normalized using the protein concentration as reference and expressed as pM/mg/ml protein [16].

### Statistical analysis

Differences between groups were analysed by the following methods: thrombus size, one-way analysis of variance (ANOVA) followed by Tukey’s multiple comparison test; TF activity, non-parametric unpaired *t*-tests with Welch’s correction; human aPL activity in mouse serum, Mann-Whitney test; and total human IgG in mouse serum, one-way ANOVA followed by Dunn’s multiple comparison tests. *P*-values < 0.05 denote a statistical difference between groups. Analysis was carried out with GraphPad Prism version 5.0 (GraphPad Software, La Jolla, CA, USA).

## Results

### Binding properties of aDI-rich and aDI-poor fractions

**Fig. 1:**
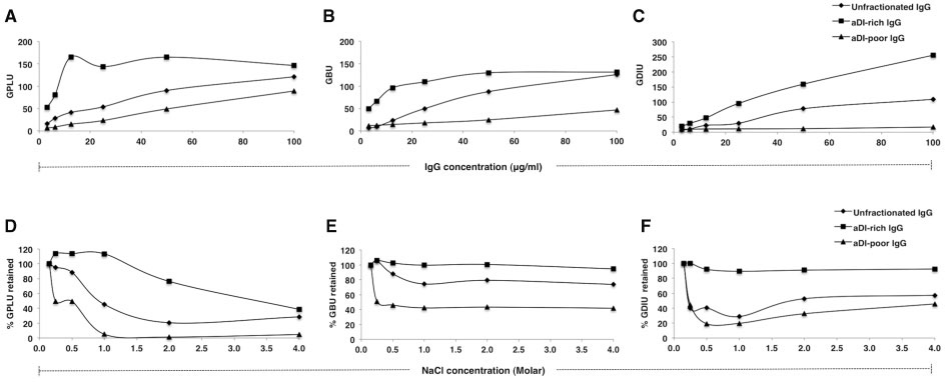
Binding properties of purified IgG fractions to cardiolipin, β_2_GPI and DI Purified IgG from unfractionated serum, aDI-rich and aDI-poor IgG from fractionated serum were tested at 100 µg/ml for (**A**) aCL, (**B**) aβ_2_GPI and (**C**) aDI activity in a dose-dependent manner. Results are expressed in IgG phospholipid units (GPLU), β_2_GPI units (GBU) and DI units (GDIU), respectively. The avidity of purified IgG (**D**) to CL, (**E**) β_2_GPI and (**F**) DI was determined by incubating IgG (at 100 µg/ml) with increasing NaCl concentrations (0.15 M original concentration in PBS, 0.25 M, 0.5 M, 1 M, 2 M and 4 M). Results are shown as a percentage of aPL activity retained, where 100% represents activity at 0.15 M NaCl. β_2_GPI: β2-glycoprotein I; DI: domain I; aDI: anti-DI; aβ_2_GPI: anti-β_2_GPI.

aCL, aβ_2_GPI and aDI titres for APS serum were >96 GPLU, 93.9 GBU and 50.2 GDIU, respectively. As illustrated in Fig. 1, purified IgG (100 µg/ml) from unfractionated serum was highly positive in all three assays, with values >96 GPLU, >100 GBU and 89.4 GDIU. aDI-rich IgG maintained high activity against all three antigens (>96 GPLU, >100 GBU, >100 GDIU); as expected, aDI activity was enriched compared with unfractionated IgG. Conversely, aDI-poor IgG confirmed reduced binding to these antigens (89.3 GPLU, 46.8 GBU, 17 GDIU); the reduction in aDI activity was by far the greatest (Fig. 1A–C). Both aDI-rich and aDI-poor IgG retained aCL, aβ_2_GPI and aDI activity above the cut-off values for positivity in each assay. All samples from healthy controls were negative in all three assays.

We determined the avidity of both aDI-rich and aDI-poor fractions for CL, β_2_GPI and DI, comparing results with unfractionated IgG (Fig. 1D–F). In all three assays the activity of aDI-poor IgG was reduced by ∼50% when NaCl was marginally increased from 0.15 to 0.25 M. In contrast, aDI-rich IgG retained >90% aβ_2_GPI and aDI activity even at 4.0 M NaCl; aCL activity was reduced, but only at ≥2.0 M NaCl. Thus, although the aDI-poor fraction retained high aCL and aβ_2_GPI activity, these aPLs were of low avidity compared with the high avidity aPL in the aDI-rich fraction.

Mice injected with aDI-rich IgG had significantly higher aPL titres in all three assays compared with mice that received aDI-poor IgG (mean activity of the aDI-rich group: 39.5 GPLU, 40.1 GBU, 16.6 GDIU; aDI-poor group: 9.3 GPLU, 13.3 GBU, 4.7 GDIU) (*n* = 9 mice/group, *P* < 0.001 in all cases). Circulating human IgG was confirmed to be present in all mice. Human IgG levels did not differ significantly between mice injected with aDI-rich and aDI-poor IgG samples (data not shown).

### In vivo thrombus-generation results

**Fig. 2:**
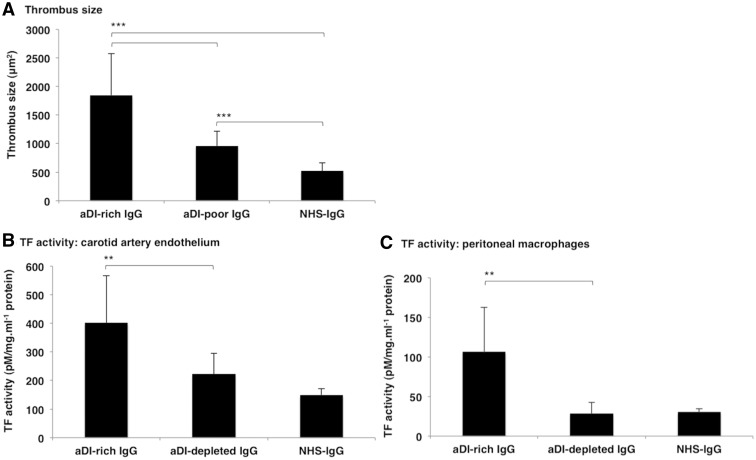
Assessing *in vivo* thrombus generation and *ex vivo* TF activity (**A**) Significantly larger thrombi were generated in the presence of aDI-rich IgG compared with either aDI-poor IgG or NHS-IgG. Significantly larger thrombi were also noted with aDI-poor IgG compared with NHS-IgG. Results are shown as mean thrombus size (µm^2^) with s.d. error bars. ***P < 0.0001. Significantly greater TF activity was seen in (**B**) carotid artery endothelial cells and (**C**) peritoneal macrophages isolated from mice treated with aDI-rich IgG compared with both aDI-poor IgG and NHS-IgG. Results are shown as the mean TF activity (pM/mg/ml) with s.d. error bars. **P < 0.01. TF: tissue factor; aDI: anti-domain I; NHS: normal human serum.

aDI-rich IgG induced significantly larger thrombi *in vivo* [mean thrombus size 1848.1 μm^2^ (s.d. 729.2) compared with aDI-poor [960.5 μm^2^ (s.d. 258.6)] or NHS-IgG [525.1 μm^2^ (s.d. 136.2)] (*P* < 0.0001 in both cases) (Fig. 2A). The aDI-poor fraction retained some prothrombotic potential, as the thrombi in these mice were significantly larger compared with mice treated with NHS-IgG (*P* < 0.0001).

### Ex vivo tissue factor activity

Both APS-derived IgG preparations increased carotid TF activity compared with NHS-IgG [mean TF activity 149.0 pM/mg/ml (s.d. 21.1)], by a factor of 2.4-fold for aDI-rich IgG [402.5 (s.d. 165.2)] and by 1.4-fold for aDI-poor IgG [223.1 (s.d. 71.8)]. However, treatment with aDI-rich IgG resulted in significantly enhanced carotid TF activity compared with aDI-poor IgG (1.6-fold difference, *P* = 0.005; Fig. 2B).

In peritoneal macrophages, treatment with aDI-rich IgG greatly enhanced TF activity by a factor of 3.5-fold compared with aDI-poor IgG [106.9 (s.d. 55.5) *vs* 28.5 (s.d. 14.1), respectively; *P* = 0.003]. aDI-poor IgG did not produce any increase in macrophage TF activity compared with NHS-IgG [30.6 (s.d. 4.1); Fig. 2C].

## Discussion

Previously we showed that recombinant human DI could inhibit the ability of polyclonal human IgG from a patient with APS to cause thrombosis or to enhance TF activity in the same *in vivo* mouse model that is described here [9]. In this report we used polyclonal IgG from a different patient with APS. In all three outcome measures, aDI-rich IgG significantly enhanced prothrombotic ability *in vivo* compared with aDI-poor or NHS-IgG. These findings are consistent with our hypothesis that the ability of human APS-derived IgG to cause thrombosis in mice is concentrated in the aDI-rich fraction. Notably the aDI-poor fraction retained some prothrombotic capacity, which may be due to (some) residual aDI activity, retained aβ_2_GPI activity (presumably binding to epitopes in DII–V) or caused by other prothrombotic IgG antibodies such as anti-prothrombin (PT) or anti-PT/phosphatidylserine [17].

Limitations of this work are that a single patient’s sample was used and that we were unable to compare the effects of aDI-rich IgG with IgG affinity-purified for binding to another region of β_2_GPI. Our expression system is currently designed to produce DI only, however, in the future it may be possible to carry out a similar experiment to test IgG specific for different domains. Our hypothesis predicts that enrichment of IgG specific to other β_2_GPI domains would not enhance the ability to cause thrombosis—all such non-aDI antibodies should be present in the aDI-poor sample used in this study.

The identification of DI as the key antigenic region for pathogenic IgG aPL [4, 5, 7] paved the way for studies reporting the association of circulating aDI with APS [8] and the ability of recombinant DI to inhibit aPL-induced thrombosis *in vivo* [9]. Importantly, a synthetic monoclonal IgG aDI (MMB2) was very recently been shown to cause complement-mediated thrombosis and fetal loss *in vivo* [18], while IgG recognizing a peptide derived from the human DI sequence induced monocyte and endothelial cell activation and bound Toll-like receptor 4 (TLR4) *in vitro*. In fact, the DI-derived peptide shared sequence homology with an epitope on TLR4 [19]. Both complement and TLR4 are historically implicated in aPL-stimulated pathogenesis and these two studies are the first to suggest that specifically aDI can drive cell damage via TLR4 and/or complement activation.

Designing a test for the detection of aDI [17] could have both diagnostic and prognostic value, allowing us to assess the susceptibility of a patient to develop an APS-related clinical manifestation. In addition, creating therapeutic agents that target aDI could offer a novel approach for treating APS [20]. Our results have provided direct evidence that human aDI is prothrombotic *in vivo*, thus emphasizing the importance of measuring circulating aDI for APS diagnosis and further highlighting the therapeutic potential of recombinant DI as a decoy for pathogenic aPL.

**Rheumatology key messages**

- First direct evidence that aDI IgG from APS patients is prothrombotic *in vivo.*
- aDI-poor IgG partially retained *in vivo* prothrombotic ability.
- aDI-rich IgG significantly enhanced endothelial and macrophage tissue factor activity compared with aDI-poor IgG.
